# Cluster Randomized Trial of Text Message Reminders to Retail Staff in Tanzanian Drug Shops Dispensing Artemether-Lumefantrine: Effect on Dispenser Knowledge and Patient Adherence

**DOI:** 10.4269/ajtmh.14-0126

**Published:** 2014-10-01

**Authors:** Katia Bruxvoort, Charles Festo, Admirabilis Kalolella, Matthew Cairns, Peter Lyaruu, Mitya Kenani, S. Patrick Kachur, Catherine Goodman, David Schellenberg

**Affiliations:** Department of Global Health and Development, Department of Infectious Disease Epidemiology, and Department of Disease Control, London School of Hygiene and Tropical Medicine, London, United Kingdom; Impact Evaluation Thematic Group, Ifakara Health Institute, Dar es Salaam, Tanzania; Malaria Branch, U.S. Centers for Disease Control and Prevention, Atlanta, Georgia

## Abstract

Artemisinin combination therapies are available in private outlets, but patient adherence might be compromised by poor advice from dispensers. In this cluster randomized trial in drug shops in Tanzania, 42 of 82 selected shops were randomized to receive text message reminders about what advice to provide when dispensing artemether-lumefantrine (AL). Eligible patients purchasing AL at shops in both arms were followed up at home and questioned about each dose taken. Dispensers were interviewed regarding knowledge of AL dispensing practices and receipt of the malaria-related text messages. We interviewed 904 patients and 110 dispensers from 77 shops. Although there was some improvement in dispenser knowledge, there was no difference between arms in adherence measured as completion of all doses (intervention 68.3%, control 69.8%, *p* [adjusted] = 0.6), or as completion of each dose at the correct time (intervention 33.1%, control 32.6%, *p* [adjusted] = 0.9). Further studies on the potential of text messages to improve adherence are needed.

## Introduction

Patient adherence to treatment is an important step in ensuring the effectiveness of artemisinin-based combination therapies (ACTs) for malaria.^1^ Incomplete adherence to recommended treatment can result in poor clinical outcomes, undermine the effectiveness of case management as a tool for malaria control, and may contribute to the selection of drug-resistant malaria parasites.^2^,^3^ ACTs are first-line treatment of *Plasmodium falciparum* malaria in the public sector of most malaria-endemic countries, with patient adherence reported to range widely from 39% to 100%.^4^,^5^ Many patients seek care for malaria in the private retail sector.^6^–^9^ The proportion of private sector clients obtaining ACTs has increased over time as ACTs have become more widely known, and their price has fallen, particularly in settings where they have been subsidized by programs such as the Affordable Medicines Facility-malaria (AMFm).^10^

Although access to effective antimalarials in the private sector may have increased, drug sellers may not always provide patients with appropriate doses or advice, raising concerns about patient adherence, though evidence is very limited. Only five studies have specifically assessed patient adherence to antimalarials obtained in the private retail sector.^11^–^15^ Of these, ACTs were used only in one study by Cohen and others (2012)^11^ in Uganda, which reported 66% of patients seeking care from drug shops were adherent. As ACTs become more available in the private sector, it becomes increasingly important to understand patient adherence and the effects of interventions intended to improve adherence. Supporting interventions, such as shopkeeper training, have previously succeeded in increasing the proportion of patients who receive and complete the recommended dose of non-ACT antimalarials,^13^,^16^ but such interventions have yet to be tested on a national scale or applied to ACTs.

Mobile phones are a promising tool for the delivery of healthcare interventions as coverage of mobile networks and handset ownership increases.^17^–^19^ Text messaging, the least expensive mobile phone function, has been used in malaria control settings for commodity monitoring, disease surveillance, and pharmacovigilance.^20^ In addition, a trial in public health facilities in Kenya^21^ showed that 6 months of text message reminders improved public health workers' management of pediatric malaria by 24% points immediately after the intervention, and the improvements were sustained for at least 6 months after the intervention was withdrawn. The text message reminders were well accepted by health workers,^22^ inexpensive, and cost-effective.^23^

Given the concerns over inadequate patient adherence to ACTs delivered through the private retail sector, and the potential benefit of text-message interventions to enhance adherence, we designed and completed a cluster randomized trial in southern Tanzania to assess the effect of text message reminders to drug shop workers on patient adherence to artemether-lumefantrine (AL). We also evaluated the effect of text messages on dispenser knowledge and advice.

The private retail sector in Tanzania is an important source of treatment of malaria,^24^,^25^ and ACT availability in such outlets increased after the implementation of AMFm in 2010. Another key intervention in Tanzania's private sector has been the creation of accredited drug dispensing outlets (ADDOs) by the Tanzania Food and Drug Administration (TFDA) to improve regulation of drug shops and quality of medicines. ADDOs are drug shops that have been upgraded through a process of training and certification and are allowed to sell a limited number of prescription-only drugs, including some antibiotics and ACTs.^26^,^27^

## Methods

### Study setting.

The study was conducted in Mtwara, a rural region in southeastern Tanzania with 35.5% of the population in the lowest national wealth quintile.^28^ Prevalence of malaria among children 6–59 months of age in Mtwara was 17.4% in the 2011–2012 HIV/AIDS and Malaria Indicator Survey^29^ and 23% in a survey of patients seeking treatment at private drug shops.^30^ AL has been recommended as the first-line treatment of malaria since 2004, although it was not available in public health facilities until 2006. The recommended treatment regimen is six doses of AL over 3 days, with 1–4 tablets (20 mg artemether/120 mg lumefantrine) per dose depending on the weight/age band. National guidelines state that the second dose should be taken 8 hours after the first dose, followed by the remaining doses morning and evening of the second and third days.^31^

In Mtwara, ADDO accreditation commenced in 2006, and officially only accredited drug stores are allowed to function. In reality, a large number of non-accredited shops exist because of a lack of training, unpaid fees, or administrative delays. These shops are tolerated by regulating authorities and considered “prospective ADDOs.” Before AMFm, ACTs were not commonly available in ADDOs in Mtwara, and dispenser training on ACTs was limited, but ACT availability significantly increased after AMFm implementation, with 88% of ADDOs stocking ACTs in Mtwara in August 2011.^32^ To support AMFm roll out, TFDA offered a 1 day refresher training that included treatment of malaria with ACTs to dispensers with previous nursing training in Mtwara in August 2011.

### Sample size calculations.

We based the sample size for this two arm cluster randomized trial on data from the public sector in Tanzania, where patient adherence to AL was 65–98%(Khatib R, unpublished data).^33^–^35^ We assumed lower adherence to AL obtained at ADDOs in Mtwara (60%) and powered the study to detect a 15% point increase in the intervention arm. We wanted to recruit a small number of patients per cluster to reduce the potential bias caused by increasing community awareness of the study's objectives. Assuming a coefficient of variation of 0.25, 80% power, 5% significance, and 20% loss to follow-up, 13 patients from 36 outlets in each arm were required, a total of 468 patients per arm.

### Selection of study ADDOs.

**Figure 1.**
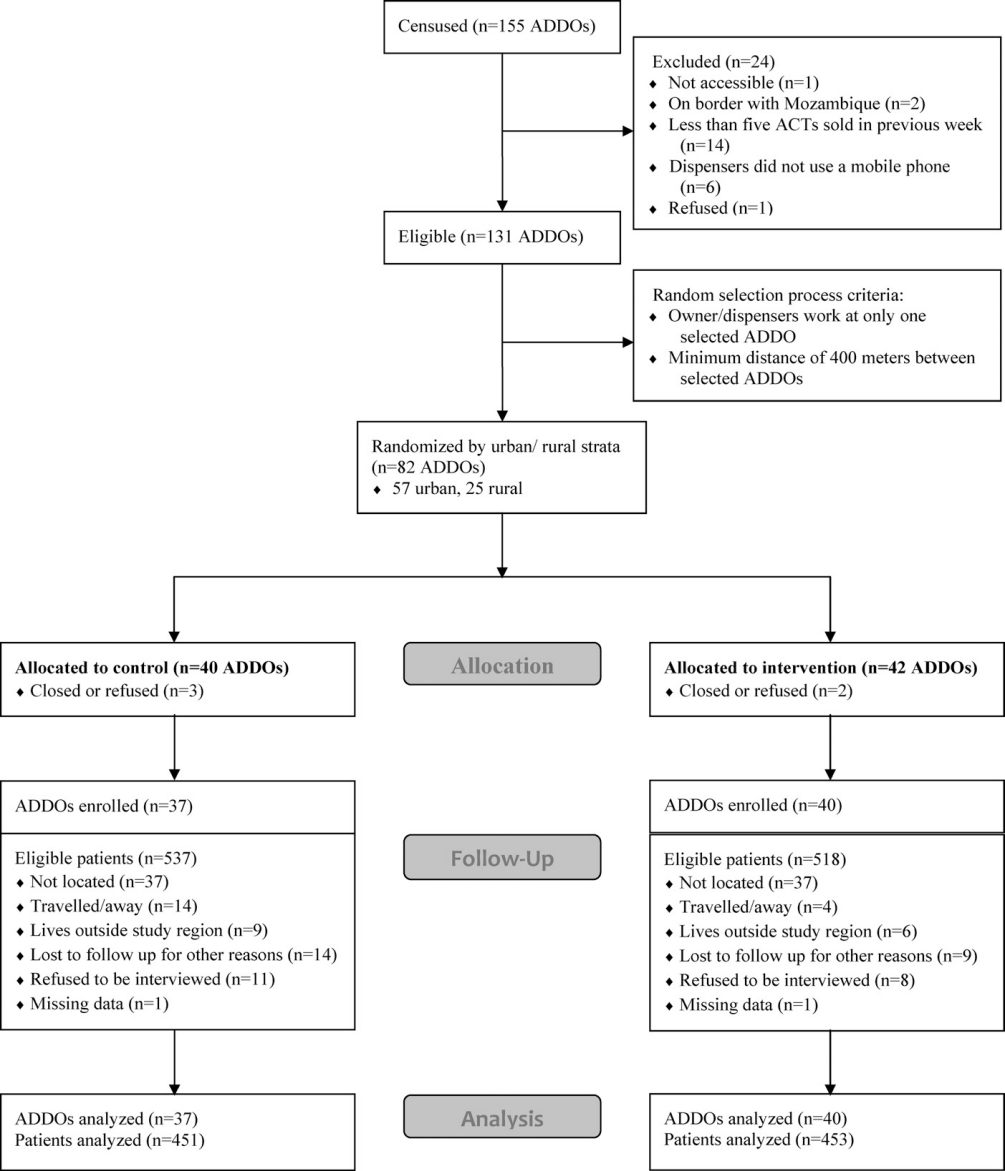
Consort-like style flow diagram of trial.

In May 2012, we conducted a census of all drug shops in Mtwara (ADDOs or prospective ADDOs). Data were collected on the characteristics of owners and dispensers, ACT stocks and sales, and global positioning system (GPS) coordinates. ADDOs were excluded from the sampling frame if they had sold fewer than five antimalarial treatments in the previous week, no dispensers used a mobile phone, the shop was located on the border with Mozambique or was not accessible, or the owner refused to participate (Figure 1). The final sampling frame consisted of 131 ADDOs. The 82 ADDOs were selected sequentially at random, with any ADDOs within 400 m of a selected ADDO, or any ADDOs where staff from a selected ADDO also worked, removed from the sampling frame. The selected ADDOs were then stratified by location in urban or rural wards, and the intervention was randomly allocated to 29 of 57 urban ADDOs and 13 of 25 rural ADDOs.

### Intervention design.

**Figure 2.**
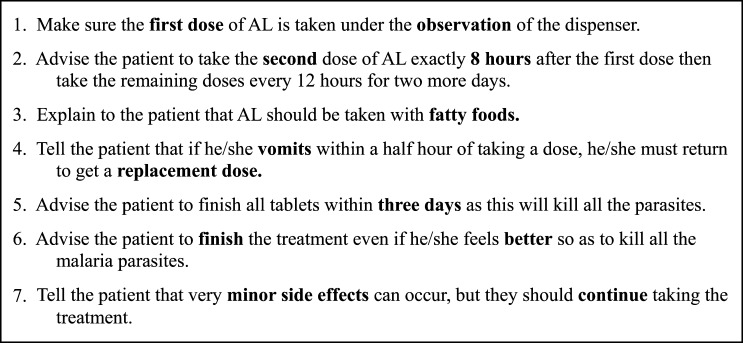
Content of text messages sent to dispensers in the intervention arm.

We designed seven content messages about advice that dispensers should provide when dispensing AL (Figure 2). The messages were derived from the government refresher training booklet and reflected the recommended practices for dispensing AL. Messages were pilot tested in ∼20 ADDOs in a semi-urban district outside of Dar es Salaam. Dispensers at these ADDOs were sent each potential message in turn and asked to explain their understanding of the meaning and relevance of each message. Dispensers were also asked if they would find receipt of the messages helpful, how often they would like to read the messages, and whether complementary components such as quotes or questions would encourage reading. Phrasing and frequency of the messages were adjusted based on feedback received during the pilot.

Before sending the first message, the 42 ADDOs in Mtwara randomized to the intervention arm were visited to invite participation and collect an updated list of mobile numbers for all dispensers. Messages began in July 2012 and were sent in random order once per day Monday–Friday for the first 4 weeks, followed by once per day Monday, Wednesday, and Friday for the next 10 weeks. Messages were written in Swahili and scheduled in advance on an automated platform, with each message paired with a different complementary component each time to promote interest. Complementary components consisted of inspirational quotes or proverbs or, once per week, a quiz question on message content that earned correct respondents free air time (500 TSH or $.30). Over the 14-week period, 49 messages were sent to each of 60 dispensers, and detailed delivery reports were kept for each message.

### Data collection.

From September through November 2012, dispensers at ADDOs in the intervention and control arms were visited by study supervisors and given a standard introduction about study objectives. To limit patients' awareness of our primary interest in adherence, which could have led to a biased assessment, dispensers were told we were studying how patients chose to treat fever and would visit some, but not all, patients at their homes. They were asked to fill out a registration form for all patients purchasing any treatment of fever, including the day and time of their ADDO visits, the patients' names, the drugs purchased, and a description of where the patients lived. Dispensers were provided with blister packs of AL that they could then sell to patients needing treatment of malaria. Study staff visited ADDOs every day to check and collect registration forms for 1–3 weeks, or until 12–15 patients purchasing AL were registered if quicker.

Eligible patients who obtained AL were identified from the registration forms and assigned patient identification numbers (recorded on follow-up forms). Patients were followed up ∼68–72 hours after their ADDO visit, according to a predefined schedule, and all attempts to locate and interview patients were recorded. Where written informed consent was given, patients or their caregivers were asked about demographic and socioeconomic characteristics, treatment-seeking history, illness symptoms, detailed information about each dose of AL taken and the advice provided by the ADDO dispenser. Blister packs were requested for a pill count, and blood samples were collected for a blood smear and a malaria rapid diagnostic test (mRDT) (Pf-specific from ICT Diagnostics, Cape Town, South Africa). Blood smears were stained in the field and transported to the Ifakara Health Institute, where they were double-read by two microscopists blinded to results from each other and the mRDT, with discrepant results read by a third microscopist.

Adherence was defined in two ways.^5^ Patients were considered to have “verified completed treatment” if they reported taking all doses by the time of the follow-up visit and a pill count verified that no pills remained in the blister pack, if available. The second, more stringent definition included a time component, based on patient reports of the time each dose was taken using the Swahili times of day: “alfajiri” (early morning), “asubuhi” (morning), “mchana” (afternoon), “jioni” (evening), “usiku” (night), and “usiku sana” (late night). Patients were considered to have “verified timely completion” if they took the second dose at the Swahili time of day corresponding with 8 hours after the first dose, and then took each of the remaining doses at the Swahili time of day corresponding with 12 hours after the previous dose, verified by the absence of pills in the blister pack. For ease of reading, we hereafter refer to these definitions as “completed treatment” and “timely completion.”

After completion of patient interviews, dispensers working at the study ADDOs were interviewed on ADDO characteristics, demographics, receipt of study text messages, and advice they would give to patients when dispensing AL. To assess knowledge corresponding with message content, we used an open-ended question, e.g., “I would like to ask about which advice you think you should provide to a person of any age taking treatment for malaria. For the following topics (e.g., “when to take the second dose” or “what to do with the pills if the patient feels better,” etc.) tell me if advice on this topic is important or not, and if so, what advice you would provide.” Responses were recorded verbatim and evaluated by the study leader using predetermined criteria based on message content.

### Data entry and analysis.

All patient and dispenser interview data were collected using personal digital assistants, and data extracted from study forms (census, registration, and follow-up forms) were double entered into Microsoft Access databases (Microsoft Corp., Redmond, WA). Data were analyzed in Stata 11.0 (Stata Corporation, College Station, TX). Primary outcomes were analyzed by intention-to-treat. Comparison of adherence was based on a *t* test of the proportion adherent in each cluster. A list of potentially important confounders was identified a priori consisting of ADDO accreditation certificate, number of customers purchasing ACTs in the previous week, dispenser medical qualification, dispenser training on ACTs, patient age, and patient education. Adjustment for variables on this list found to be unbalanced between arms was performed by fitting a logistic regression model to the individual data and performing analysis on the aggregated residuals, as described by Bennett and colleagues.^37^

### Ethics.

All questionnaires, consent forms, and other study documents were translated into Swahili and piloted before use. Written informed consent was collected from dispensers before census, patient registration and interview, and from patients or their caregivers prior to interview. The study protocol was approved by the ethical review boards of Ifakara Health Institute and London School of Hygiene and Tropical Medicine. The Centers for Disease Control and Prevention (CDC) investigators provided technical assistance in design and analysis but were not engaged in data collection. The trial is registered with Current Controlled Trials, ISRCTN83765567.

## Results

Figure 1 shows the trial profile. Of the 82 randomized ADDOs, 37 from the control arm and 40 from the intervention arm participated in the study. The number of registered patients eligible for follow-up was 537 in the control arm and 518 in the intervention arm, with ∼15% of patients in each arm lost to follow-up. Most outlets were in urban wards (70% in both arms), had a single dispenser, and had at least some ACTs in stock on the day of interview (Table 1). Of 51 dispensers in the control arm and 59 in the intervention arm, ∼80% in both arms were female and had a low-level medical qualification, mostly nurse assistants (Table 2). Though low in both arms, more ADDOs in the control arm compared with the intervention arm were able to show an accreditation certificate (43% versus 20%, respectively). However, the difference in the percentages of dispensers that had received training on ACTs was not as pronounced (69% in the control arm versus 60% in the intervention arm), though the median year of training was more recent in the control arm (2011 versus 2009).

Characteristics of patients were well balanced between arms (Table 3). A high percentage of patients (36% in the control arm and 38% in the intervention arm) had sought care before attending the study ADDO, with most patients going to a kiosk or general shop and only 7% of patients in the control arm and 4% in the intervention arm going to a public health facility. Approximately 90% reported symptoms of fever or headache, and approximately half had upset stomachs or nausea. Based on an mRDT taken at the time of follow-up, 28% in the control arm and 25% in the intervention arm tested positive, with only 1.4% and 1.6%, respectively, testing positive by study blood smear. (Some degree of discrepancy is expected because of the persistence of HRP2 detected by the mRDT.)

**Figure 3.**
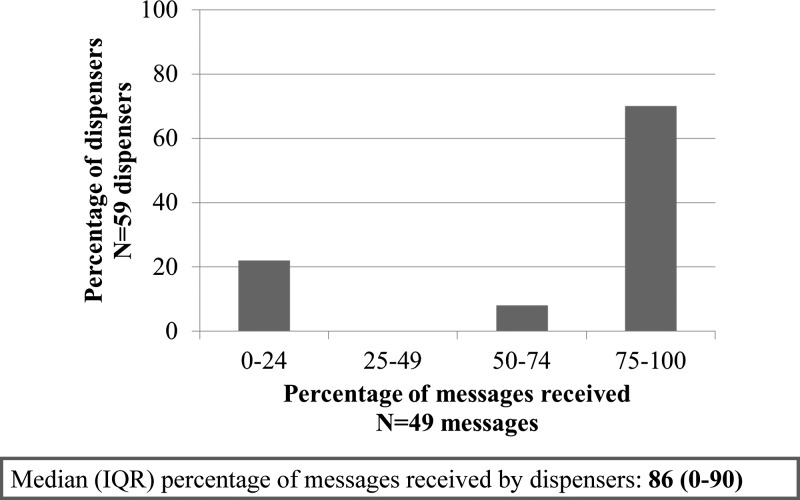
Percentage of text messages received by dispensers in the intervention arm.

Seventy percent of dispensers received at least 75% of the text messages. The median percentage of messages received was 86%, with 20% of dispensers receiving no messages (Figure 3). Table 4 presents results of the dispenser interviews on knowledge of advice to provide patients when dispensing AL. Dispensers in the intervention arm reported slightly better knowledge of the correct AL regimen for adults in the intervention arm compared with the control arm (90% versus 78%; adjusted prevalence ratio [aPR] = 1.2 (95% confidence interval [CI]: 0.95, 1.5); *p* [adjusted] = 0.0748), though knowledge of the correct regimen for a child aged four weighing 20 kg was lower than for adults in both arms (75% versus 64%; aPR = 1.2 [0.85, 1.7]; *p* [adjusted] = 0.2). Dispenser knowledge was considerably higher in the intervention arm than the control arm on advice to take AL with fatty food (60% versus 20%; aPR = 3.4 [95% CI: 1.6, 7.1]; *p* [adjusted] < 0.0001) and to continue to take AL if minor side effects occur (68% versus 43%; aPR = 1.6 [95% CI: 1.0, 2.4]; *p* [adjusted] = 0.0188). However, surprisingly high dispenser knowledge (87–99%) was recorded in both arms on advice to complete treatment even if feeling better, advice to return to the ADDO or go to a health facility if the condition worsens, and advice to take the second dose after 8 hours (Table 4). Knowledge on advising patients to take a replacement dose in case of vomiting within half an hour of taking a dose was lower (55% versus 50%), with no difference observed between arms.

Table 5 shows that ∼60% of patients in both arms reported being told how to take AL correctly, with similar percentages reporting being told to take the second dose after 8 hours and to complete treatment even if feeling better, indicating that dispensers were providing some advice even in the absence of the intervention. However, no differences were found between control and intervention arms for any piece of advice. Less than 5% of patients in both arms reported being told about vomiting, side effects, or taking AL with fatty food, and < 10% in both arms took the first dose of AL at the ADDO.

**Figure 4.**
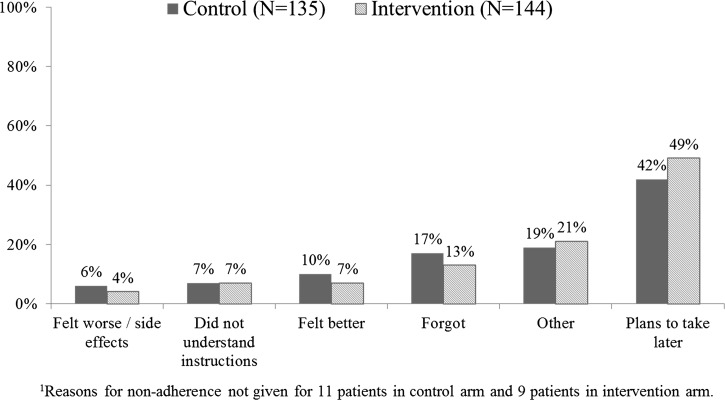
Reasons given by patients/caretakers for not completing treatment.

There was no difference in patient adherence between arms (Table 6). Completed treatment was 70% in the control arm and 68% in the intervention arm (adjusted risk ratio [aRR] = 0.96 [95% CI: 0.82, 1.1]; *p* [adjusted] = 0.6), with a similar percentage of patients adherent to each of the four age-appropriate blister packs and no important differences between arms. The mean number of doses taken by non-adherent patients was four in both arms, and the most common reported reasons for non-adherence included planning to take the medication later, forgetting to take the tablets, feeling better, and other reasons (Figure 4). Timely completion was much lower, with 33% of patients in both arms taking all doses at appropriate times. A per protocol analysis, excluding patients attending ADDOs where at least one dispenser did not receive any messages, had no impact on results.

## Discussion

We have reported results from a cluster randomized trial of a text message intervention directed at drug shop dispensers to improve patient adherence to ACTs in Tanzania. The intervention increased dispenser knowledge of some components of advice to provide patients when dispensing AL, but knowledge of other components was already very high in the absence of the intervention. The improvements in knowledge did not translate into an increase in information patients reported receiving, even though patients commonly reported receiving some advice. There was no difference in adherence of patients to the ACT regimen between the arms.

Adherence by completed treatment was < 70% to ACTs obtained from ADDOs, comparable to the 66% adherent by the same definition in the study by Cohen and others (2012)^11^ in the private retail sector in Uganda. However, timely completion was only 33% in our study, indicating that even patients who complete treatment may do so with poor adherence to the recommended schedule. Both of these results are comparable to reported adherence to ACTs obtained from public health facilities, where studies under real life conditions have found adherence of 64–77% for completed treatment verified by pill count and 39–75% for timely completion verified by pill count,^5^ including one study from Tanzania.^35^ Two other studies from public health facilities in mainland Tanzania using different definitions and methods have reported higher adherence (88.3% and 90%).^33^,^34^

Although the text message intervention targeting dispensers was not effective at improving patient adherence, there was a marked increase in dispenser knowledge of advising patients to take AL with fatty foods or milk and to continue AL even if minor side effects occurred. However, knowledge in both arms was surprisingly high, particularly on advising patients to take the second dose after 8 hours, to complete treatment even if feeling better, and to seek further care if the condition worsens. This could reflect the recent ADDO trainings in Mtwara, raising the possibility that the intervention's impact could have been different in the absence of recent training.

Knowledge did not necessarily result in the provision of advice, even though some advice was provided. For example, 98% of dispensers in both arms knew it was important to advise patients to complete treatment even if feeling better, but only 60% of patients reported receiving this advice. Other advice was much less commonly provided, with < 5% of patients in both arms reporting being advised on what to do in case of minor side-effects or vomiting, even though dispenser knowledge of this advice was much higher. This may be because the dispensers did not deem the advice helpful to their business or to the patients, as it could heighten a negative perception about the effects of their products. Dispensers may have also perceived that clients were in a hurry or not receptive to advice. Alternatively, patients or caretakers may not have recalled the advice given to them several days before. Exit interviews or mystery shopper surveys may have been useful in assessing whether advice was communicated, but these methods also have limitations, such as greater potential for a Hawthorne effect and ethical challenges.

We intentionally avoided telling dispensers that the purpose of our study was to improve patients' adherence. Although dispensers receiving text messages were aware that the content focused on advising patients about the correct use of AL, we did not mention the objective to dispensers in either arm to avoid this information being relayed to patients, who might then change their behavior because they expected their adherence to be monitored.^5^ However, if this intervention were to be scaled up outside the study context, one might include greater emphasis on adherence and its value in communications with dispensers, which might in turn increase the likelihood that they would provide appropriate advice.

The evaluation of patient adherence relied on self-reported data from patients or their caregivers, which may be subject to recall and social desirability bias. We inspected blister packs for the 80% of patients who could provide them and identified only 10 patients (1%) that had reported completing all doses but had pills remaining. On the other hand, patients may have removed pills from the packaging to consume at a later time. Even though Swahili times of day, based on sunrise and sunset, were used to assess timely completion, patients or their caregivers may not have remembered when each dose was taken or may have provided the expected responses to avoid being seen as negligent.

A similar text message intervention targeting health workers in public health facilities in Kenya found significant improvements in health worker case management of pediatric malaria.^21^ The primary outcome measure by Zurovac and colleagues included completion of four treatment tasks (e.g., prescribing AL) and at least four of six dispensing and counseling tasks, of which the biggest improvements were seen in giving the first dose at the health facility and advising patients to take the second dose after 8 hours, take each dose after a meal, and what to do in case of vomiting. Although we found strong evidence in the intervention arm of improved dispenser knowledge of advice to take each dose with a fatty meal, we recorded high dispenser knowledge in both arms of advice to take the second dose after 8 hours and no difference between arms in knowledge of advice on what to do in case of vomiting. We also recorded < 10% of patients in either arm taking the first dose of AL at the ADDO, even though drinking water was available at many ADDOs. The contrasts between our findings and those of Zurovac and colleagues could reflect the private drug shop setting, as we found patients' relatives often seek care at ADDOs on behalf of patients, in contrast to public health facilities where patients themselves must be present for a clinical exam. Health workers in public health facilities may also be more accustomed to taking on advisory roles than dispensers in private drug shops and less concerned with making a profit.^37^

Interventions involving training of dispensers in the private retail sector, although limited in number, have improved dispenser knowledge across a range of diseases and settings, but the impact of improved knowledge on dispenser and patient behavior has been mixed.^37^,^38^ Even fewer studies have reported effects of an intervention targeted at retail dispensers on patient adherence to antimalarial drugs. One study from 1998–2001 in Kilifi, Kenya found that trained shopkeepers were willing to take on an advisory role, resulting in both increases in advice and the proportion of patients taking adequate doses of chloroquine and sulfadoxine pyrimethamine or sulfadoxine-pyrimethamine.^14^ Although receiving instructions has been associated with patient adherence to antimalarials in several studies in the public and private sectors,^11^,^39^,^40^ other factors might also influence patient adherence, including patient education, higher socioeconomic status, treatment-seeking behavior, understanding the instructions, knowledge and perceptions of the illness or of the drug, and satisfaction with information received or with the drug.^5^

The private retail sector is likely to continue to be an important source of treatment of malaria and there is a need to maximize patient adherence to ACTs. Given the effectiveness of text message reminders on health worker case management in Kenya and the low cost of this intervention, there is potential for further evaluations of text message interventions targeted at dispensers in the private retail sector to improve dispenser knowledge, advice provided, and patient adherence, particularly in settings where dispensers have not received recent training on malaria. Such interventions should ensure message content addresses gaps in dispenser knowledge and would benefit from additional research on dispenser readiness to provide advice, and client receptivity to their advice. There should also be further consideration of how best to design the evaluation so that dispensers are motivated to communicate the importance of adherence without biasing study results.

The double gap between dispenser knowledge and providing advice and then patients receiving advice and being adherent may also call for other interventions to enhance adherence. Text message reminders to patients have been shown to be a low-cost approach to improve patient adherence to antiretroviral therapy for HIV^41^–^43^ and have been used in two recent studies to increase patient adherence to malaria test results and treatment (Goldberg J, unpublished data).^44^ However, scaling up a text message intervention targeted at malaria patients in Tanzania would require an increase in personal mobile phone use among patients most at risk of malaria,^45^ as only about half of the households in rural areas own a mobile phone,^29^ and the phone may be shared among household members. In contrast, nearly all dispensers censused in Mtwara regularly used at least one mobile phone.

Other interventions that have been shown to improve patient adherence to antimalarial drugs include packaging and community education.^46^ ACTs are now mostly available in factory packaged unit dose packs blister packs with illustrated instructions, therefore additional room for improvement may be limited. One possible modification could be improved instructions in local languages. Community education through communication campaigns could be helpful in emphasizing the importance of taking all doses, but it may be challenging to communicate the details of when and how to take each dose to the general population. Finally, the introduction of mRDTs in the private sector might have positive implications for patient adherence, especially if also combined with dispenser advice.^5^

## Conclusion

Text message reminders improved some aspects of dispenser knowledge of advice to provide to patients when dispensing AL in the private sector. However, patients in the intervention arm were not more likely to report receiving improved advice and did not have higher adherence than patients in the control arm. Adherence to AL among patients in both arms was suboptimal, highlighting the need for studies evaluating other interventions to improve adherence to ACTs obtained in the private retail sector.

## Figures and Tables

**Table 1:** Characteristics of accredited drug dispensing outlets (ADDOs)

	Control	Intervention
Number (*N*)	37	40
Number urban (%)	26 (70%)	28 (70%)
Median number of dispensers per ADDO (range)	1 (1–3)	1 (1–4)
Number with one or more trained medical staff (%)*	36 (97%)	37 (92%)
Number with any ACTs in stock on day of interview (%)	35 (95%)	40 (100%)
Number with all four weight-based packs in stock on day of interview (%)	11 (30%)	14 (35%)
Median number of customers purchasing ACTs in last 7 days (range)	13 (0–82)	19 (0–147)
Number with ADDO accreditation certificate (%)	16 (43%)	8 (20%)
Number with drinking water available in ADDO (%)	30 (81%)	37 (93%)

* Medical staff is defined as pharmacists, pharmacist assistants, medical doctors, assistant medical doctors, clinical officers, assistant clinical officers, midwives, nurses, nurse assistants, and laboratory technologists. Most were nurse assistants or nurses.

ACTs = artemisinin-based combination therapies.

**Table 2:** Characteristics of dispensers (post-intervention)

	Control	Intervention
Total number of dispensers	53	59
Number interviewed (*N*)	51	59
Male (%)*	10 (20%)	13 (22%)
Age (%)*		
Under 35 years of age	18 (35%)	23 (39%)
35–49 years of age	23 (45%)	17 (29%)
50 years and above	10 (20%)	19 (32%)
Number with a medical qualification (%)*†	44 (86%)	47 (81%)
Socioeconomic status (%)‡§		
1st quintile (most poor)	9 (18%)	13 (22%)
2nd quintile	12 (24%)	10 (17%)
3rd quintile	9 (18%)	13 (22%)
4th quintile	8 (16%)	14 (24%)
5th quintile (least poor)	12 (24%)	9 (15%)
Number that had attended training on ACTs (%)*	35 (69%)	35 (60%)
Median year of training (range)	2011 (2005–2012)	2009 (2001–2012)

* Data missing for one dispenser in intervention arm.

† Medical staff is defined as pharmacists, pharmacist assistants, medical doctors, assistant medical doctors, clinical officers, assistant clinical officers, midwives, nurses, nurse assistants, and laboratory technologists. Most were nurse assistants or nurses.

‡ Data missing for one dispenser in control arm.

§ Wealth quintiles determined using a principal component analysis of sampled dispensers based on standard Demographic and Health Survey variables.

**Table 3:** Characteristics of patients

	Control	Intervention
Number (*N*)	451	453
Male	240 (53%)	211 (47%)
Age*		
Under 3 years	81 (18%)	78 (17%)
3 years to under 8 years	104 (23%)	91 (20%)
8 years to under 12 years	41 (9%)	42 (9%)
12 years and above	225 (50%)	242 (53%)
Blister pack obtained		
1 × 6 (6 tablets)	109 (24%)	107 (23%)
2 × 6 (12 tablets)	95 (21%)	88 (19%)
3 × 6 (18 tablets)	50 (11%)	43 (10%)
4 × 6 (24 tablets)	197 (44%)	215 (48%)
Patient (or caregiver if patient below age 12) completed primary school†	323 (72%)	343 (76%)
Socioeconomic status‡		
1st quintile (most poor)	87 (19%)	94 (21%)
2nd quintile	99 (22%)	82 (18%)
3rd quintile	97 (22%)	84 (19%)
4th quintile	85 (19%)	96 (21%)
5th quintile (least poor)	83 (18%)	97 (21%)
Slept under any bed net the night before the follow up interview	321 (71%)	357 (79%)
Sought care before attending study ADDO	171 (38%)	163 (36%)
Median days since illness onset before seeking care at ADDO§	1	1
Symptoms¶		
Fever or headache	410 (91%)	416 (92%)
Respiratory	34 (8%)	34 (8%)
Stomach upset	220 (49%)	209 (46%)
Other∥	216 (48%)	211 (47%)
mRDT positive at follow up**	121 (28%)	108 (25%)
Blood smear positive at follow up††	6 (1.4%)	7 (1.6%)

* Age categories based on recommended age breakdown for artemether-lumefantrine (AL) blister packs in Tanzania.

† Caregiver education missing for 3 patients < 12 in intervention arm.

‡ Wealth quintiles determined using a principal component analysis of sampled patients based on standard Demographic and Health Survey variables.

§ Eleven patients in control arm and 4 patients in intervention arm did not remember the number of days after illness onset when they sought care at the study accredited drug dispensing outlets (ADDO).

¶ Percents do not add to 100% as patients experienced multiple symptoms.

∥ Includes fatigue, body aches, dizziness, shaking, convulsions, unusually-colored urine, yellow mouth/eyes/body, etc.

** Malaria rapid diagnostic test (mRDT) data missing for 12 patients in control arm and 12 patients in intervention arm.

†† Blood smear data missing for 18 patients in control arm and 13 patients in intervention arm.

**Table 4:** Dispenser knowledge of correct advice (mean of cluster summaries)

	Control* (*N* = 37) % (SD)	Intervention* (*N* = 40) % (SD)	Adjusted prevalence ratio (95% CI)†	Adjusted *P* value†
Proportion that gave correct advice on:				
Correct AL regimen for adult‡	78.4 (38.3)	90.0 (33.7)	1.19 (0.95, 1.49)	0.075
Correct AL regimen for a child (4 years and 20 kg)‡	63.5 (46.6)	74.6 (40.4)	1.20 (0.85, 1.70)	0.2
Take with fatty food	20.3 (38.1)	60.0 (45.6)	3.41 (1.63, 7.12)	0.0001
Continue treatment if minor side effects occur	42.8 (42.8)	67.5 (45.2)	1.58 (1.03, 2.42)	0.019
Return to ADDO or go to a health facility if condition worsens	91.0 (25.3)	100.0	1.04 (0.84, 1.31)	0.7
Take second dose after 8 hours	86.9 (32.2)	97.5 (15.8)	1.11 (0.93, 1.32)	0.1
Take replacement dose in case of vomiting within half hour of taking dose	49.5 (45.7)	55.0 (46.4)	1.20 (0.77, 1.86)	0.4
Complete treatment even if feeling better	98.6 (8.2)	98.8 (7.9)	1.07 (0.88, 1.30)	0.5

* Total number of dispensers interviewed was 51 in the control arm and 59 in the intervention arm.

† Adjusted for accredited drug dispensing outlet (ADDO) accreditation, number of customers at ADDO purchasing artemisinin-based combination therapies (ACTs) (< 20 vs. 20 or more), dispenser medical qualification, and training on ACTs

‡ To be considered correct, responses had to identify artemether-lumefantrine (AL) as first-line treatment and specify that six doses should be taken, with each dose consisting of four pills (adult) or 2 pills (child 4 years of age). Dose intervals considered correct included (A) taking a dose morning and evening for 3 days or (B) taking the second dose 8 hours after the first dose and the remaining doses 12 hours apart (or morning and evening for the next 2 days).

**Table 5:** Patient report of advice received from dispenser (mean of cluster summaries)

	Control* (*N* = 37) % (SD)	Intervention* (*N* = 40) % (SD)	Adjusted prevalence ratio (95% CI)†	Adjusted *P* value†
Explained correct dose regimen‡	60.6 (21.2)	62.9 (21.5)	1.00 (0.84, 1.20)	0.9
Told to take second dose after 8 hours	64.2 (19.6)	63.0 (20.2)	1.00 (0.87, 1.15)	0.9
Told to complete treatment even if feeling better	61.3 (25.3)	59.3 (27.3)	0.94 (0.76, 1.17)	0.5
Told not to give drug to anyone else or save for future illnesses	41.3 (23.3)	35.1 (25.1)	0.88 (0.65, 1.18)	0.4
Told to return to ADDO or go to a health facility if condition worsens	34.1 (20.5)	35.0 (22.4)	1.01 (0.75, 1.35)	0.9
Told to take replacement dose in case of vomiting	3.3 (8.9)	3.2 (3.2)	1.48 (0.56, 3.90)	0.5
Told about possible side effects	2.8 (5.1)	2.0 (5.1)	0.60 (0.13, 2.77)	0.4
Told to take each dose with fatty food or milk§	2.2 (8.4)	4.2 (9.7)	1.70 (0.40, 7.24)	0.4
First dose was observed at ADDO	5.4 (9.1)	6.9 (12.7)	1.32 (0.63, 2.76)	0.5

* Total number of patients interviewed was 451 in the control arm and 453 in the intervention arm.

† Adjusted for accredited drug dispensing outlet (ADDO) accreditation, number of customers at ADDO purchasing artemisinin-based combination therapies (ACTs) (< 20 vs. 20 or more), dispenser medical qualification, and training on ACTs

‡ To be considered correct, responses had to include the correct number of pills per dose for blister pack obtained, two doses per day, and 3 days duration (or 4 days to account for artemether-lumefantrine [AL] obtained late on Day 1)

§ If taking with any food or milk is considered correct, percentages increase to 60.2 (22.1) in the control arm and 58.8 (26.0) in the intervention arm, *P* = 0.8)

**Table 6:** Patient adherence (mean of cluster summaries)

	Control* (*N* = 37) % (SD)	Intervention* (*N* = 40) % (SD)	Adjusted prevalence ratio (95% CI)†	Adjusted *P* value†
Completed treatment‡	69.8 (20.9)	68.3 (23.4)	0.96 (0.82, 1.12)	0.6
1 × 6 (6 tablets)	68.2 (33.3)	73.4 (30.9)	1.04 (0.81, 1.33)	0.7
2 × 6 (12 tablets)	62.0 (33.4)	70.7 (40.5)	1.10 (0.85, 1.43)	0.5
3 × 6 (18 tablets)	73.0 (35.7)	62.5 (39.7)	0.86 (0.66, 1.13)	0.4
4 × 6 (24 tablets)	73.8 (23.1)	67.8 (29.1)	0.89 (0.74, 1.08)	0.2
Timely completion§	32.6 (18.4)	33.1 (21.6)	1.01 (0.76, 1.36)	0.9
1 × 6 (6 tablets)	37.2 (36.7)	26.4 (34.5)	0.67 (0.35, 1.28)	0.2
2 × 6 (12 tablets)	25.7 (24.9)	33.1 (36.6)	1.32 (0.86, 2.00)	0.4
3 × 6 (18 tablets)	35.5 (41.3)	37.9 (41.4)	1.02 (0.57, 1.81)	0.9
4 × 6 (24 tablets)	38.1 (27.6)	34.8 (29.3)	0.90 (0.57, 1.41)	0.6

* Total number of patients interviewed was 451 in the control arm and 453 in the intervention arm.

† Adjusted for accredited drug dispensing outlet (ADDO) accreditation, number of customers at ADDO purchasing artemisinin-based combination therapies (ACTs) (< 20 vs. 20 or more), and patient education (patient or caregiver completed primary school).

‡ Completed treatment unknown for three patients in the control arm and two patients in the intervention arm.

§ Timely completion unknown for 10 patients in the control arm and 11 patients in the intervention arm.
